# Enhancement of Anaerobic Digestion to Treat Saline Sludge from Recirculating Aquaculture Systems

**DOI:** 10.1155/2015/479101

**Published:** 2015-08-02

**Authors:** Guo-zhi Luo, Niannian Ma, Ping Li, Hong-xin Tan, Wenchang Liu

**Affiliations:** ^1^College of Fisheries and Life Science, Shanghai Ocean University, Shanghai 201306, China; ^2^Shanghai Collaborative Innovation Center for Aquatic Animal Genetics and Breeding, Shanghai 201306, China; ^3^Research and Development Center of Aquacultural Engineering of Shanghai, Shanghai 201306, China

## Abstract

The effectiveness of carbohydrate addition and the use of ultrasonication as a pretreatment for the mesophilic anaerobic digestion of saline aquacultural sludge was assessed. Analyses were conducted using an anaerobic sequencing batch reactor (ASBR), which included stopped gas production attributed to the saline inhibition. After increasing the C : N ratio, gas production was observed, and the total chemical oxygen demand (TCOD) removal efficiency increased from 75% to 80%. The TCOD removal efficiency of the sonication period was approximately 85%, compared to 75% for the untreated waste. Ultrasonication of aquaculture sludge was also found to enhance the gas production rate and the TCOD removal efficiency. The average volatile fatty acid (VFA) to alkalinity ratios ranged from 0.1 to 0.05, confirming the stability of the digesters. Furthermore, soluble chemical oxygen demand (SCOD), VFA, and PO_4_^3−^ concentrations increased in the effluents. There was a 114% greater gas generation during the ultrasonication period, with an average production of 0.08 g COD/L·day^−1^.

## 1. Introduction

The scope of digestion in fish is limited because a relatively large fraction of feed remains undigested and is then excreted [1]. In a properly managed farm, between 11 and 40% of applied feed has been estimated to accumulate as sludge [2]. To date, much of the focus has been on the treatment of dissolved waste nutrients from aquaculture systems [3]. Less attention has been paid to solid wastes, which are often discharged as sludge, creating a significant amount of unused nutrients.

Although anaerobic digestion (AD) is commonly used to stabilize municipal, industrial, and agricultural wastes, it is a novel approach for the treatment of sludge produced in recirculating aquaculture systems (RAS) [4]. Conventional anaerobic digesters have been used to treat aquaculture sludge in previous studies. These digesters include continuous flow stirred tank reactors (CSTR) [5, 6] and upflow anaerobic sludge blanket (UASB) reactors [7–9]. Recent digester developments, such as the anaerobic sequencing batch reactor (ASBR), allow for the treatment of high-solids waste streams in high-rate systems [10].

Sludge pretreatment is required to rupture the cell wall and facilitate the release of intracellular matter into the aqueous phase. This increases biodegradability and enhances anaerobic digestion, with lower retention time and with higher biogas production [11]. Extensive research has been conducted throughout the world to establish the most effective yet economically feasible pretreatment technology for enhancing sludge digestion. One such method, ultrasonication, is an emerging and promising mechanical disruption technique [11–13].

The suitability of waste sludge for anaerobic digestion largely depends on its physical, chemical, and biological characteristics. Generally, the reported optimal C : N ratio for methanogenesis and AD of sludge ranges from 21 to 32 [14]. Typically, fish sludge is characterized by its low C : N ratio compared to other animal production or industrial wastewater processes [7]. This may contribute to anaerobic digestion not being more widely commercialized [15].

Because sludge is not collected in the traditional aquacultures (in ponds, flow-through systems, or net pens), information about aquaculture sludge treatment is lacking [4]. The treatment of saline organic wastewater from seawater aquacultures and brackish aquacultures is a matter of special importance. There have been few studies addressing the effects of sonication pretreatment and added carbon sources on saline aquacultural sludge for digestion in an ASBR. In this study, we added glucose and utilized ultrasonication as a pretreatment method to enhance anaerobic digestion in a laboratory-scale ASBR in order to effectively treat saline sludge from a recirculating aquaculture system.

## 2. Materials and Methods

### 2.1. Experimental Anaerobic Sequencing Batch Reactors (ASBRs)

**Figure 1:**
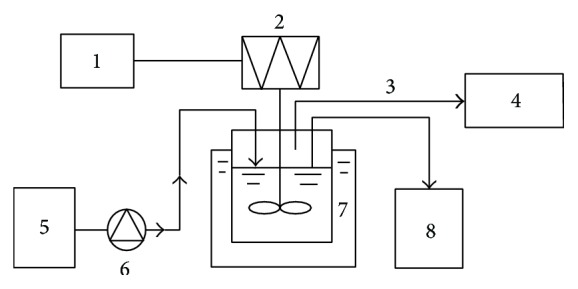
Schematic diagram of the anaerobic sequencing batch reactor (ASBR). 1 timer; 2 stirrer; 3 biogas; 4 aluminum bags; 5 influent tank; 6 peristaltic pump; 7 water-bath; and 8 effluent tank.

The experiment was conducted at a mesophilic temperature of 35 ± 1°C in oroglas (poly-methyl acrylic acid methyl ester) ASBR reactors (Figure 1), which were placed in a water-bath and stirred continuously at 150 rpm (round per minute). Reactors were 14 cm in diameter and 26 cm high, with an operating volume of approximately 4 L (a work volume of 3 L and a headspace of 1 L). The ASBR operates in a cyclic batch mode, with four distinct phases per cycle. The four phases are feeding (15 min), reacting (46.5 h), thickening (1 h), and drawing (15 min). The thickening and drawing phases are the key steps in the ASBR operation. The thickening phase accumulates sludge, as the solids remain within the reactor. Each reactor was loaded with 3 L of anaerobic sludge. The reactors were sampled and fed manually, using a double siphon and a tube through the digester lid. Biogas was collected in 10-L aluminum bags. The experiment was conducted in the dark.

### 2.2. Fish Farming Sludge (Substrate)

Sludge was collected from a recirculating aquaculture system (RAS) used for the production of* Scortum bacoo* at the Center for Recirculating Aquaculture Systems of Shanghai Ocean University (Shanghai, China). The sludge was collected once a day over a three-month period. Fish were fed on a commercial pellet diet (moisture 3%; crude protein 45.0%; crude lipid 8.0%; Ca 1.8%; P 1.5%; lysine ≥ 2.9%; and methionine 1.4%) (Suzhou Tong Wei Special Feed Co., Ltd., Wujiang, China). The feed conversion ratio (kg feed used kg fish produced^−1^) was 2.0. The composition of the sludge is shown in Table 1. The sludge was kept frozen at −18°C until the experiment began.

### 2.3. Experimental Set-Up

A period of 165 days was considered adequate for establishing the performance of untreated aquaculture sludge. The salinity was gradually increased from 0 to 10.5‰ over a period of 75 days in order to enable probable adaptation of the biomass. The related results can be found in another study [16]. The hydraulic retention time (HRT) was 20 days. The organic loading rate (OLR) was 0.39–0.41 g COD L^−1^ d^−1^. When the outlet saline level reached 8.7‰, gas production was not observed. In this study, we analyzed the addition of carbohydrates and use of ultrasonication as a pretreatment method for the enhancement of saline aquaculture sludge anaerobic digestion.

### 2.4. Experimental Design

The digester enhancement process operated for 55 days (Table 1). Approximately five different operating regimes can be distinguished: period 1 (days 1–10), without an external source; period 2 (days 11–20), we increased the C/N ratio of the influent to 15 by adding glucose; period 3 (days 21–30), we increased the C/N ratio of the influent to 20; period 4 (days 31–40), without an external carbon source, and with influent the same as period 1; period 5 (days 41–55), ultrasonication pretreatment of the digester influent.

When feeding the digester (every two days), the stirrer was switched off and the digester contents were allowed to settle for approximately 60 minutes. 300 mL of liquor was then removed through the effluent port at the top of the digester for analysis. An equivalent volume of aquaculture waste, as mentioned above, was then added via the influent port. Finally, the stirrer was again switched on. Raw aquaculture wastes were adjusted to approximately 10.5‰ by dilution with saline water and to approximately 7.0 by the addition of sodium hydrogen carbonate before use.

When sonication was applied, the aquaculture waste effluents were ultrasonicated for 10 minutes using a 120 w sonication bath operating at a frequency of 50 kHZ. This time period was chosen because it corresponded with previously reported methods [17, 18].

### 2.5. Analytical Methods

Samples were analyzed for pH, alkalinity, and salinity using a YSI556 meter (YSI Incorporated 1725, Yellow Springs, OH, USA). Total ammonium nitrogen (TAN) and phosphorous (PO_4_^3−^) were determined according to APHA standards (2005). SCOD and TCOD were determined as described by Jirka and Carter [19]. Prior to the SCOD and TCOD analyses, the samples were diluted to less than 700 mg COD L^−1^ [6]. Gas production was manually measured by emptying the gasbags via suction. The process used an acid sodium sulfate solution (DIN 38 414-S8, 1985) and a tightly sealed demijohn of 25 L. The collected liquid was then weighed. The weighed amount was corrected for the pressure difference caused by the height difference between the fluid levels in the demijohn and the collection can, according to previously published methods [5, 6]. The concentrations of volatile fatty acids (VFA) were analyzed using a gas chromatograph equipped with a megabore capillary column and a flame ionization detector, with nitrogen as the carrier gas.

## 3. Results and Discussion

**Figure 2:**
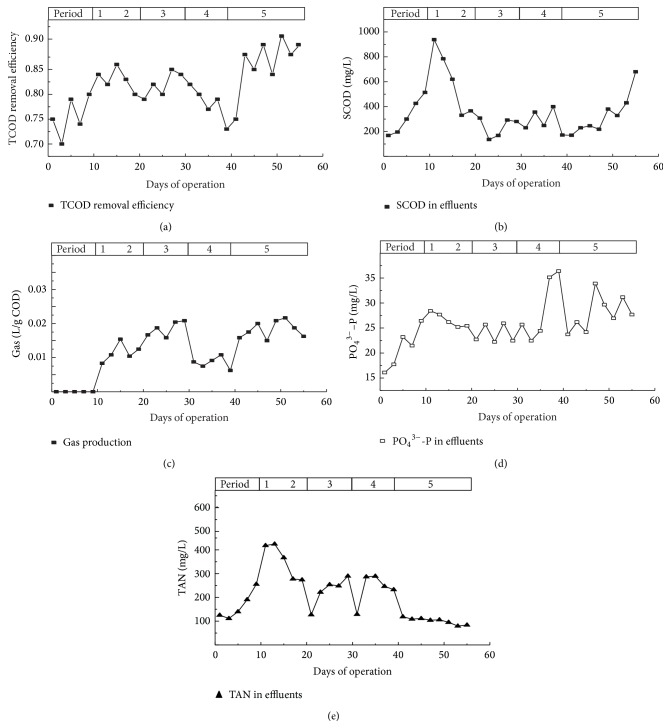
(a) Total chemical oxygen demand (TCOD) removal efficiency; (b) soluble chemical oxygen demand (SCOD) removal efficiency; (c) gas production; (d) PO_4_^3−^ in effluents, and (e) total ammonia nitrogen (TAN) in effluents of the anaerobic sequencing batch reactor (ASBR) during the experimental period.

Aquaculture waste feed supplied to the digester had a TCOD concentration of 8.0 ± 0.4 g L^−1^. The addition of glucose to the influent increased the TCOD removal efficiency from 75% to 83% for period 2 and 82% for period 3 (Figure 2(a)). There were no appreciable increases observed between period 3 and period 2, suggesting that a C/N ratio of 15 was appropriate for the reactors. The high effluent SCOD during period 2 can be attributed to the reactors ineffectively processing the added glucose. The effluent SCOD decreased after the reactors were adapted (Figure 2(b)).

The TCOD removal efficiency for the sonication period was approximately 85%, compared to 75% for untreated waste period 1 (Figure 2(a)). Ultrasonic pretreatment solubilized extracellular matter and extracellular polymeric substances (EPS) [17, 20], as reflected by an SCOD increase of 19% and VFA increase of 53% in the reactors (Figures 2(b) and 2(c)).  Figure 2 illustrates that the ultrasound resulted in the solubilization of organic material from the solid to the aqueous phase, thus making the sludge more biodegradable. The sludge SCOD increased due to solubilization of the solid phase matter. In addition, organic matter and EPS concentrations increased in the aqueous phase. Therefore, the SCOD of the effluents can be used as a parameter to evaluate the sludge disintegration [11].

VFA concentrations were less than 150 mg L^−1^ for the entire digestion period (not presented), indicating a stabilized degradation process [21]. The ultrasonication of digester feed led to an increase in VFA concentration, corresponding to the findings of Wang et al. [22].

The gas production of the reactors was observed after the addition of glucose and increased with the C/N ratio.  Figure 2(d) shows the cumulative production of biogas throughout the study. Between days 30 and 40 (Period 4), there was a vast decrease in gas production, which corresponded with the ending of the carbon addition. These results suggest that it is necessary to add carbon during the anaerobic digestion process when treating saline sludge from recirculating aquaculture systems. There was a 114% greater generation of gas during the sonication period, with an average production of 0.08 g COD/L day^−1^ (days 41–58).

The removal of soluble phosphorus was monitored throughout the study period (Figure 2(d)). The PO_4_^3−^ in the effluents of the reactor did not significantly vary after the addition of glucose. However, there was a noticeable increase in the effluent PO_4_^3−^ concentrations. This may be attributed to the release of phosphorous from the solid matrix during aquaculture waste ultrasonication.

Ultrasonication increases organic nitrogen and ammonia concentrations in sludge samples [23]. McDermott et al. showed that there was a significant increase in TAN removal during the feed pretreatment period due to ultrasonication [17]. It is unclear why the TAN in effluents in this study declined, with an average concentration of 101 ± 1.5 mg L^−1^ during the sonication period (Figure 2(e)), compared with 184.5 mg L^−1^ in period 1, 350 mg L^−1^ in periods 2-3, and 23.8 mg L^−1^ in period 4.

The VFA effluent concentrations were under 140 ± 3.5 mg L^−1^ for the entire digestion period. The alkalinity was between 1500 and 3000 mg L^−1^. The VFA/alkalinity ratio can be used as a measure of process stability. When this ratio is less than 0.3–0.4, the process is considered to be operating favorably, without acidification risk [21]. The average VFA to alkalinity ratios in this study were 0.1–0.05, confirming the digester's stability.

The effluent alkalinity declined with the addition of glucose and pretreatment by sonication. This suggests that there was a noticeable anaerobic digestibility improvement when the C/N ratio increased and sonication was utilized for feed pretreatment [24].

## 4. Conclusions

The pretreatment of aquaculture sludge via ultrasonication and carbohydrate addition improved the performance of the saline digestion process. Gas production was observed, and the TCOD removal efficiency increased from 75% to 80% after the C/N ratio was increased to 15. Aquaculture sludge ultrasonication was also found to enhance the gas production rate and TCOD removal efficiency. Furthermore, there was an increase in SCOD, VFA, and PO_4_^3−^ concentrations in the effluents.

## Figures and Tables

**Table 1:** Cultivation strategy in all five phases.

Phase	1	2	3	4	5
Operating time (d)	10	10	10	10	15
Organic loading rate (OLA) g COD (L·d)^−1^	0.39–0.41	0.39–0.41	0.39–0.41	0.39–0.41	0.39–0.41
C : N ratio	10	15	20	10	10
Ultrasonication pretreatment	No	No	No	No	Yes
